# A Landscape of Opportunities for Microbial Ecology Research

**DOI:** 10.3389/fmicb.2020.561427

**Published:** 2020-11-20

**Authors:** Cendrine Mony, Philippe Vandenkoornhuyse, Brendan J. M. Bohannan, Kabir Peay, Mathew A Leibold

**Affiliations:** ^1^UMR CNRS ECOBIO, Université de Rennes, Rennes, France; ^2^Institute of Ecology and Evolution, University of Oregon, Eugene, ORE, United States; ^3^Department of Biology, University of Stanford, Stanford, CA, United States; ^4^Department of Biology, University of Florida, Gainesville, FL, United States

**Keywords:** landscape ecology, metacommunity, microbial assembly-rules, dispersal, plant microbiota, human microbiota, animal microbiota

## Abstract

Microbes encompass tremendous biodiversity, provide support to all living forms, including humans, and play an important role in many ecosystem services. The rules that govern microorganism community assembly are increasingly revealed due to key advances in molecular and analytical methods but their understanding remain a key challenge in microbial ecology. The existence of biogeographic patterns within microbial communities has been established and explained in relation to landscape-scale processes, including selection, drift, dispersal and mutation. The effect of habitat patchiness on microorganisms’ assembly rules remains though incompletely understood. Here, we review how landscape ecology principles can be adapted to explore new perspectives on the mechanisms that determine microbial community structure. To provide a general overview, we characterize microbial landscapes, the spatial and temporal scales of the mechanisms that drive microbial assembly and the feedback between microorganisms and landscape structure. We provide evidence for the effects of landscape heterogeneity, landscape fragmentation and landscape dynamics on microbial community structure, and show that predictions made for macro-organisms at least partly also apply to microorganisms. We explain why emerging metacommunity approaches in microbial ecology should include explicit characterization of landscape structure in their development and interpretation. We also explain how biotic interactions, such as competition, prey-predator or mutualist relations may influence the microbial landscape and may be involved in the above-mentioned feedback process. However, we argue that the application of landscape ecology to the microbial world cannot simply involve transposing existing theoretical frameworks. This is due to the particularity of these organisms, in terms of size, generation time, and for some of them, tight interaction with hosts. These characteristics imply dealing with unusual and dependent space and time scales of effect. Evolutionary processes have also a strong importance in microorganisms’ response to their landscapes. Lastly, microorganisms’ activity and distribution induce feedback effects on the landscape that have to be taken into account. The transposition of the landscape ecology framework to microorganisms provides many challenging research directions for microbial ecology.

## Introduction

Microorganisms represent by far the largest fraction of biodiversity (Curtis and Sloan, 2005). There are 100 million times as many bacteria in the oceans (13 × 10^2^8) as there are stars in the known universe […] [Editorial Nature Reviews in Microbiology, (No authors listed, 2011)]. The amazing abundance of microorganisms on earth plays a central role in the biogeochemical cycles of elements (Curtis and Sloan, 2005), affects soil fertility, organic matter decomposition and carbon storage. Microorganisms are also required to sustain all living macroorganisms, including humans (Curtis, 2006), as they are involved in the nutrition, health, reproduction and behavior of their hosts (Curtis, 2006; Boulangé et al., 2016; Vuong et al., 2017). They consequently ensure the majority of ecosystem services provided to our society (Philippot et al., 2013; Vandenkoornhuyse et al., 2015). However, microorganisms display a substantial spatial heterogeneity (Box 1, see reviews Etterma and Wardle, 2002; Green and Bohannan, 2006; Franklin and Mills, 2007; Bahram et al., 2015). This raises questions about how the distribution of microbes depends on different components of community assembly, its link with niche theory and coexistence mechanisms, and how community assembly is linked to the functions and functioning of these microbial ecosystems.

**Main definitions.**

**Species:** a group of organisms that are able to exchange genes or interbreed, and create fertile offsprings. The species is the principal taxonomic unit of classification.

**Habitat:** the area characterized by a given set of environmental variables (abiotic and biotic factors) required by a species for survival, growth and reproduction.

**Spatial heterogeneity:** Non-random distribution of species or individuals within an area. Spatial heterogeneity can be related to landscape heterogeneity or a property of the population.

**Microbial Landscape:** Elements hosting microbial community spatially distributed and with interactions among them (exchange of individuals, energy and matter). It can be both structural landscapes (set of patches characterized by their environmental conditions) or biotic landscapes (set of hosts of various species, genotypes and ages). Microbial landscape can be seen from kilometric to centimetric scales.

**Landscape heterogeneity:** Differences in landscape elements in terms of composition and configuration. Heterogeneity is reached while there are a complex composition and configurations of landscape elements in the landscape. Landscape homogenization is the process leading to a decrease in landscape heterogeneity.

**Landscape composition:** Number and type of landscape elements.

**Landscape configuration:** Spatial arrangement of landscape elements. It can be related to the size, the location and the form of the habitat patches.

**Landscape fragmentation:** Habitat configuration within a landscape depending on isolation and habitat patch size. Landscape fragmentation is also the process involving the loss and the breaking apart of habitat.

**Habitat isolation:** Distance among patches of a given habitat type. Habitat isolation refers to the ability of organisms to move among habitat patches.

**Habitat amount:** Total patch area of a given habitat type. Habitat amount is linked to the carrying capacity for organisms.

**Dispersal:** Movement of organisms that has an effect on the genetic structure of populations, and communities (emigration-immigration process from one patch to another).

**Species selection:** Selection of species depending on their traits that promote their fitness in a given environment.

**Ecological drift:** Random change in demographic rates of survival and reproduction.

**FIGURE 1:**
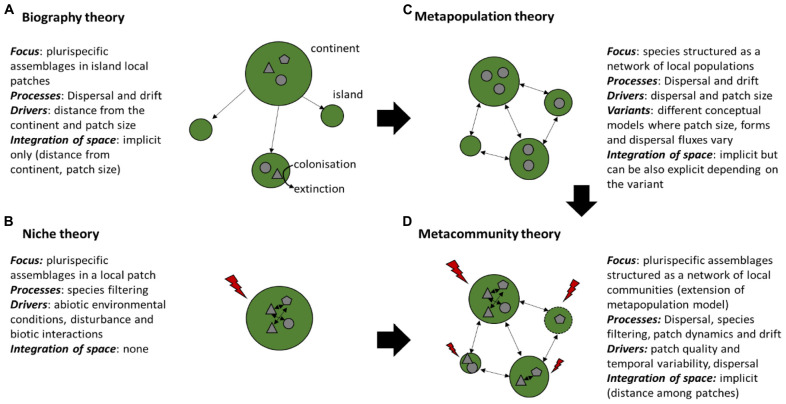
The main conceptual frameworks of species assembly: **(A)** Biogeography theory, **(B)** Niche theory, **(C)** Metapopulation theory, **(D)** Metacommunity theory. **(A,B)** consider drivers at the local patch scale, while **(C,D)** consider the network structure of populations and communities. Green forms represent habitat patches, arrows among green forms represent dispersal fluxes, arrows within green forms represent species interactions, gray geometrical forms represent species, and red lightning represent abiotic conditions. When several types of geometrical forms are present in a given patch, it means that assemblages are plurispecific (i.e., include different species). Patches that are getting disturbed are delimited with dotted lines.

Drivers of microorganism assemblages have so far mostly been analyzed at the patch scale, assuming that species niches result from the effect of the abiotic environment on species selection, disturbance or biotic interactions among microbial organisms (Niche theory, Figure 1), or with their host (Louca et al., 2018) and ignoring dispersal effects. Because microorganisms have very high reproductive capacities and short generation time, the historical view “*everything is everywhere but the environment selects*” (Baas Becking, 1934) has been accepted for a long time. The progress in resolution of microbial communities composition obtained from mass sequencing and large-scale studies of microbial distribution (see for instance Karimi et al., 2018) provided an increased number of evidences that microorganisms are much more limited in their dispersal than previously suspected (Telford et al., 2006). A framework based on large-scale biogeography has been successfully used for understanding large-scale spatial patterns of species (e.g., Martiny et al., 2006; Hanson et al., 2012; Donaldson et al., 2016; Figure 1). This framework considers that community assembly in local patches considered as “islands” results from colonization and extinction processes, both processes are related to the size and distance of the patch to a source patch (“continent”) (Figure 1). A spatially implicit approach that builds on the island biogeography theory of MacArthur and Wilson (1967) was a useful starting point to consider how dispersal can affect community assembly at the landscape level, starting at first on one species (i.e., metapopulation, Hanski, 1994; Figure 1), to assemblages with several species (i.e., metacommunity, Leibold et al., 2004; Figure 1). Metacommunities consist of sets of communities connected through dispersal. Four main processes can thus drive community variation in space, which are species selection (including both abiotic and biotic factors), speciation (analogous to mutation in population genetics), dispersal and ecological drift (Box 1; Vellend, 2010, 2016).

**FIGURE 2:**
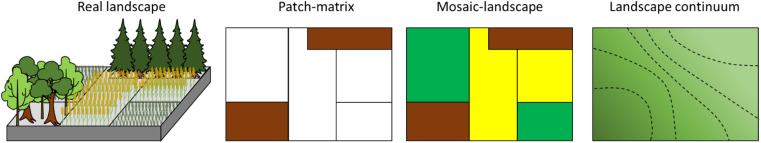
Conceptual models of landscapes. Landscapes are represented as composed of discrete patches (patch-matrix and mosaic-landscape), or as an ecological continuum (landscape continuum). Real landscape is constituted of two kinds of forest patches, grasslands (green plants) and crops (yellow plants). Patch-matrix considers only the habitat patch and not the mosaic (e.g., only the forest patches here in brown). Mosaic-landscape considers mosaic composition and habitat patches (e.g., forest patches are in brown, grasslands in green and crops in yellow). In Patch-matrix and Mosaic-landscape models, black lines represent the limits of the discrete patches. Landscape continuum considers landscapes as described through continuous environmental variables (organic matter content for instance, or cover density). In this latter model, landscape can be linked with one or several continuous variables. Dotted lines represent different levels of the environmental gradient.

**FIGURE 3:**
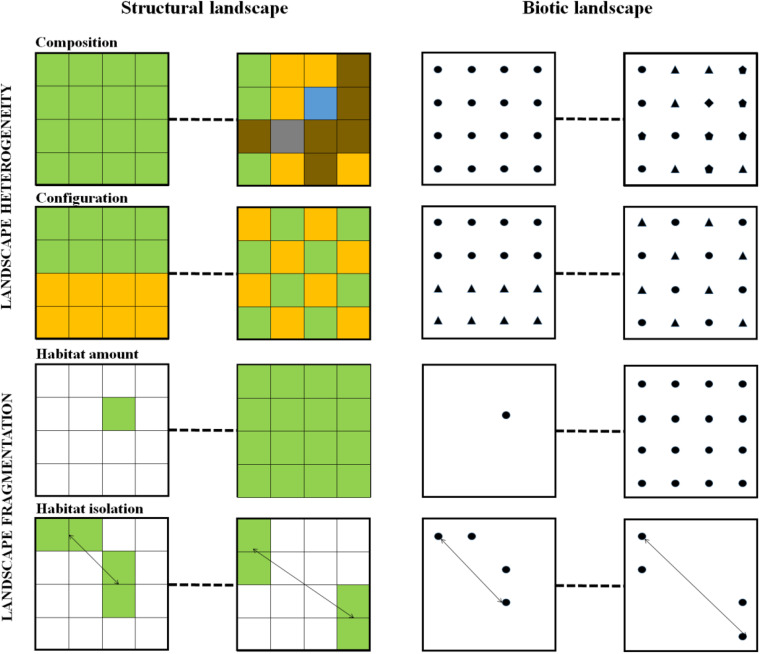
Landscape heterogeneity and fragmentation in both structural and biotic landscapes. Gradients of low to high values of these components are represented. On the left, the colors represent different landscape elements. Landscape elements can be different land covers or land uses, different anatomical sites within a body, or different environmental patches within a given anatomical site. In this example, the focus habitat is the green one, where the microbial assemblages is studied. On the right, the geometrical shapes represent the distribution of different types of hosts. Biotic landscapes are then considered as the composition and configuration of the potential hosts. Hosts can be several species, or several genotypes within species. In this example, the focus habitat is the round host type, which microbiota is studied. See Box 1 for definitions.

An alternative approach to spatial dynamics in ecology emerged some 30 years ago in the form of landscape ecology (Wiens et al., 1993; Turner et al., 2001; Fletcher and Fortin, 2018). Landscape ecology focused specifically on the explicit analysis of spatial ecological patterns and has determined the conceptualization of what a landscape is (Figure 2), and provided tools for analyzing how spatial processes influenced the assembly of biodiversity, focusing primarily on plants and animals (Box 1). Landscape structure, described through different metrics at the landscape scale (i.e., heterogeneity) or the habitat scale (i.e., fragmentation), has been shown to affect dispersal, and local habitat exploitation during an organism’s lifecycle, but also the availability of habitat for species development and movements among local patches (Figure 3 and Box 1). These two types of metrics - landscape heterogeneity and fragmentation - affect species abundance and composition (Fahrig, 2003). Landscape ecology research has, however, mostly concentrated on macroorganisms and the microbial compartment has remained understudied under this framework.

Applying landscape ecology principles to microorganisms has, until recently, been slow to develop due to our limited understanding of microbial habitat requirements, the difficulties involved in observing microorganisms movements and our limited capacity to conduct spatially extensive surveys of microbial distribution. The determination of microbial community composition is also by itself difficult given that microbial communities can be quite complex and need to be studied by mass-sequencing approaches. From the nature of the data used, the microbial species-sequence delineation is also needed and the adoption of a phylogenetic species concept (i.e., “[…] the smallest diagnosable cluster of individual organisms within which there is a pattern of ancestry and descent. […]” (Cracraft, 1983)) is implicit. After having used cutoff of sequence identity to identify Operational Taxonomic Units, recent bioinformatics advances now allow circumventing the use of this artificial cutoff (i.e., sequence-clusters (Mahé et al., 2014) and Amplicon Sequence Variants (Callahan et al., 2017)) and define taxa at a thinner grain. This better resolution in community description provides the basis for testing new ecological concepts such as landscape ecology. Application of landscape ecology to the microbial world also requires the characterization of the landscapes in which microbes live. Such landscapes can be a set of different habitat types with varying environmental conditions, but it can be the set of hosts available for microbial colonization (Figure 3). These “biotic” landscapes may then be driven by the behavior and growth of macro-organisms that are hosts for microorganisms, and be dependent on these hosts’ response to their own landscape characteristics. Lastly, microbial landscapes can be within a host, corresponding to different anatomical sites within the body, and even within each organ (Batten et al., 2007; Proctor and Relman, 2017), providing patches varying in their environmental conditions. In addition, individual microorganisms interact with nano and microscale surface features and volumes (Hol and Dekker, 2014). Unusual small-scale landscapes have then to be taken into consideration due to the very small size of microorganisms. Within these microscale landscapes as in terrestrial or aquatic environments, microorganisms’ distribution is tightly related to patch heterogeneity (Nunan et al., 2003; Vos et al., 2013). However, most authors did not ground their work in the landscape ecology framework, and it is only recently that microbial ecology developed explicit integration of landscape ecology principles for understanding the drivers of microbial distribution (Batten et al., 2007). Because of these specificities of microbes - species-definition, dispersal, response to biotic heterogeneity, and small-scale responses to environment - the transposition of the existing theoretical framework in landscape ecology for analyzing assembly rules of microorganisms is likely indirect.

The aim of the present review was to investigate how landscape ecology concepts could apply to the microbial world, to advance our understanding of this world and to show how microorganisms can be used as new models to test and extend the existing landscape ecology framework. We accounted for aquatic, terrestrial and marine ecosystems, as well as for all kinds of host-microbiota interactions from free-living microorganisms to microorganisms associated with plants, animals and humans. Viruses were excluded from the scope of the paper because of their sub-microscopic size. If there are compelling reasons about the importance of viruses for the origin of cells and diversification (Koonin et al., 2009), there is evidence against the notion that viruses are alive (Moreira and López-García, 2009). Their dispersion, genetic changes and propagation are thus determined by specific constraints not developed herein.

## From Environmental Spatial Patterns to Landscapes

### Landscape Conceptual Models

Species distribution can be related to environmental patchiness via the way abiotic (or biotic) conditions are distributed in space. The consequences of such environmental patchiness on ecological processes including species assembly can be analyzed using different conceptual models of landscapes (Figure 2). The very first, and simplistic, conceptual model derived from the island biogeographic theory, considered that identical habitat patches (i.e., corresponding to favorable niches) are embedded in a matrix of distinct non-habitat (Patch matrix model, Figure 2; Turner, 1989). In this first vision, landscapes are characterized by metrics quantifying the amount of favorable patches or their isolation, supposing that the rest of the landscape do not act on species assembly. The patch-matrix model was then rapidly extended to the mosaic landscape model (Figure 2; Wiens, 1995) by including the mosaic of habitats comprising the landscape, and that could be considered as composed of more or less favorable habitats for development (Fahrig et al., 2011). Species are indeed potentially dependent on different habitat patches for their life cycle (i.e., complementation concept, Dunning et al., 1992) as for instance for animals, which juvenile stages depend on one given habitat type and adult stages on another. They can also rely on alternative habitats for their development (i.e., supplementation concept, Dunning et al., 1992). The mosaic of patches shapes also dispersal by acting on the permeability of landscapes to species movement (Taylor et al., 1993). In this second vision, landscapes are characterized by metrics quantifying their heterogeneity in terms of patch composition, which defines the type, richness and relative abundance of the different patches (i.e., abiotic habitats or hosts types) (Box 1). Heterogeneity of configuration defines the arrangement in space of the different patches and is related to metrics measuring features such patch size, aggregation, interface types among patches (Box 1; Fahrig et al., 2011). A last conceptual model has recently emerged, the continuum model, considering that landscapes are a combination of several continuous environmental gradients (Figure 2; Fischer and Lindenmayer, 2006; Cushman et al., 2010) instead of discrete patches. For this model, metrics used to describe the landscape are continuous, and may integrate partly species response to these environmental gradients through for instance the degree of matching between the abiotic conditions and species ecological requirements.

Most microbial studies based on landscape ecology are based on the patch-matrix model, while the mosaic model is only used in particular cases where patches are very heterogenenous, or where discrete patches correspond to different hosts. Correlations between environmental factors and microbial community composition have been extensively studied (Bru et al., 2011; Vos et al., 2013; Dehkharghani et al., 2019; Martínez-Olivas et al., 2019; Muscarella et al., 2019). Even though they generally imply the continuum conceptual model, these patterns have not been very well connected to the underlying theory behind landscape conceptual models. We review the existing evidence of landscape effects in the section “Effects of Landscape Mosaic Heterogeneity and Habitat Fragmentation on Microorganisms” below.

### Spatial and Temporal Scales in Community Structure Across the Landscape

A major question in landscape ecology is the scale of effect (Jackson and Fahrig, 2012; Miguet et al., 2016), i.e., at which scale the landscape variables has to be measured for a better prediction of the relationships between landscape variables and the biological response (e.g., species richness or diversity). This scale of effect is assumed to be related to the scale at which the species perceive and interact with the landscape. It can be analyzed both in space and over time. The standard landscape scale for macro-organisms ranges from hundreds to thousands of square meters, in relation with landscape patchiness and organism’s dispersal range. The response of macro organisms can be measured over years, decades or even centuries in relation with landscape dynamics and an organism’s life span. The scales of response by microorganisms is likely to differ because of their small size and short life span.

#### Spatial Scales of Microbial Landscapes

Existing literature on microorganism assemblages based on landscape ecology deals with large-scale landscapes (i.e., kilometric landscapes). Large-scale approaches are appropriate given the dispersal distance of many free-living organisms, thanks to the role of vectors such as wind, particles or water fluxes. For instance, the hydrological connectivity in a river floodplain system (i.e., at the kilometric scale) influences the abundance and productivity of bacteria (Luef et al., 2007). The distribution of host-associated microorganisms responds at similarly, large spatial scales given the ability of their hosts to disperse over such a distance and be a vector for these microorganisms. Such a large spatial distance has long been appropriate to assess the dynamics and consequences of animal and plant diseases (Yuen and Mila, 2015) as fungi and bacteria pathogens are mostly spread by the long-distance vectors such as wind, human and animals mentioned above. Landscape epidemiology has developed on this background in order to help predict disease risk and disease propagation from landscape structure (see reviews by Holdenrieder et al., 2004; Suzan et al., 2012).

Although the conceptual framework of landscape ecology has not been explicitly used, a number of studies have demonstrated that microbial assemblages can be shaped by spatial heterogeneities that occur at very small spatial scales. For example, experimental studies that manipulated very small-scale differences in resource supply can produce correspondingly small landscape-induced microbial community changes (Keymer et al., 2006). In an experiment using microfluidic device where patchy and continuous landscapes were modeled, bacterial prey and predator relationships displayed different patterns: prey population in the continuous landscape progressively declined toward extinction, whereas significant stable prey population remained in the patchy landscape, indicating that microscale fragmentation significantly influenced bacterial composition and interactions (Hol et al., 2016). In response to chemical gradients, motile cells have evolved chemotaxis and chemotactic decision and behavior to reach favorable environments (Salek et al., 2019). Chemotactic velocity and performance capabilities in bacteria in response to environmental heterogeneity was habitat-of-origin dependent, seemingly higher for bacteria from the ocean in comparison to bacteria from gut for example (Son et al., 2016). More recently, landscape ecology principles were applied successfully to biotic landscapes, i.e., landscapes viewed as sets of hosts. Biotic landscape heterogeneity shaped endophytic fungal assemblages in plant roots at centimetric scales (Bittebiere et al., 2020; Mony et al., 2020a), with contrasted responses: Ascomycota depended on the floristic landscape composition through plant evenness and richness, while Basidiomycota depended on the floristic landscape configuration through host plant aggregation and connectivity (Mony et al., 2020a).

In the particular case of a microbial landscape in a host, the biological scale considered, for instance the whole body or the specific anatomical site, defines the landscape boundaries. Intra-host spatial patchiness has so far mainly been studied on human hosts and less on other organisms. For instance, the centimetric landscape mosaic of heterogeneous environmental patches has been described in different anatomical sites including the human nose, mouth, and throat, mostly with the objective to predict on microbial distribution and composition and their consequences for disease (See examples in the review of Proctor and Relman, 2017). Extending these investigations to other animal or plant hosts would be an interesting direction for future research.

#### Temporal Scales in Microbial Landscape Dynamics

Most existing work only implicitly accounts for time, for instance, when studying the microbial succession along series of past occupations of a given patch (Soledad Faggioli et al., 2019) or across the different developmental stages of a host (Bahram et al., 2015; Charbonneau et al., 2016; Chesneau et al., 2020). Explicitly accounting for the spatial scale in these temporal dynamics, i.e., analyzing the effects of temporal changes in the landscape has, however, been clearly overlooked (but see the studies on patch dynamics effect on planktonic and bacteria assemblages, Pringle et al., 1988).

Yet, landscapes change over time, at different scales ranging from hours to years, and likely modifying the dynamics of microorganism assemblages. Such landscape dynamics may be due to simultaneous changes in the land cover, land use or environmental conditions in the local patches that together form the landscape. In host-associated microbial communities, composition is strongly linked to the phenology of their host. For instance, in honey bees, the diversity of the bacterial community and in the type of genera colonizing the gut microbiota of young workers differs from that in 1-month older workers (Dong et al., 2020), likely due to changes in the diet and in the developmental environment, especially the social tasks and contacts attributed to older workers. In this particular case, the microbial landscape constituted by the distribution of individual bees is likely driven by population dynamics and their resulting host age-distribution and associated social interactions. Such population-dynamics driven biotic landscapes need to be investigated in many host species, including plants, animals and humans, where microbial community succession has been demonstrated to depend on the developmental stage of the host. These changes can happen in days as is the case for insects (e.g., Duguma et al., 2015; Dong et al., 2020), to several years in the case of hosts with longer life span (e.g. examples in Bahram et al., 2015; Dzidic et al., 2018).

In the particular case of landscapes within hosts, time also plays an important role in changes in the type and spatial arrangement of the environmental patchiness. For instance, children’s teeth erupt at different developmental stages–from milk teeth to permanent teeth, and the sequence of eruption of the different classes of teeth (molars, incisors, canines), leads to changes in the spatial distribution of the microorganisms that inhabit the oral cavity (Dzidic et al., 2018). Teeth patch dynamics indeed had an impact on the occurrence of new patch types to be colonized and on local abiotic factors that induced modifications in species dominance, even within the same genus (for instance *Streptococcus* species, Carlsson et al., 1975). Changes in landscape heterogeneity at a finer temporal scale have also been reported to result from slight modification of saliva fluxes, or local inflammation patches in the oral cavity, likely inducing changes in bacterial composition within the course of a single day.

Overall, microbial modifications can be induced by landscape characteristics at different time and space scales, potentially in interaction, and in many situations nested within each other.

### Nested Spatial and Temporal Scales

In landscape ecology, nested relationships among spatial scales and to a lesser extent among temporal scales within a landscape are accounted for in the hierarchy theory (Allen and Starr, 1982; O’Neill et al., 1986), which states that ecosystem processes are organized in discrete scales of interaction. Wu and Loucks (1995) proposed including time in this theoretical framework through the hierarchical patch dynamics concept that integrates patch dynamics in the hierarchy theory and provides a conceptual framework for analyzing interactions among spatial and temporal scales in landscapes.

**FIGURE 4:**
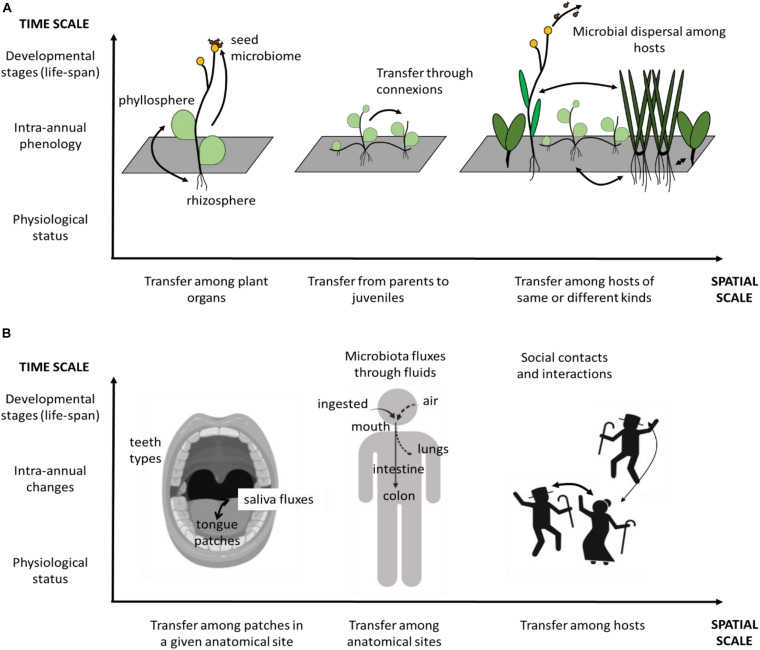
Examples of nested scales in space and time of microbiota distribution. **(A)** microbiota-associated with host plants, micro-scale landscape corresponds to the organ partitioning within the individual plant. Meso-scale landscape corresponds to the distribution of individual plants at the local scale due to the effect of neighborhood and vegetative multiplication. Large-scale landscapes correspond to microbial transmission among individual plants and through seed dispersal. **(B)** Microbiota-associated with humans. Micro-scale landscape corresponds to the different patches within an anatomical site, here, the oral cavity. Meso-scale corresponds to the different anatomical sites within the human body, connected through dispersal. Large scale corresponds to social interactions and contacts among different humans. In both examples, microbiota changes occur at different temporal scales: short scale of an hour or a day (e.g., changes in physiological status), medium scale of a year (e.g., intra-annual phenological stages), and long scale covering decades (e.g., developmental stages in an individual life cycle). These temporal scales affect all the spatial scales illustrated in the figure.

The nested relationship among spatial and temporal scales of landscape probably also applies to microorganisms. Here we provide two examples of such potential nestedness (Figure 4). First example is linked with microorganism distribution in the human body. Such distribution depends on the local landscape of a given anatomic site, in the nose, for instance (Proctor and Relman, 2017) or in the oral cavity (Proctor et al., 2018). However, microorganisms can also disperse across anatomic sites (von Eiff et al., 2001) as the nasal and oral cavities both drain into the pharynx, which ultimately connects through the trachea to the lungs or through the esophagus to the stomach. Microbiota can then disperse among human hosts depending on types of social contacts and behavior: for instance, microbiota exchanges between oral cavities depend on human partners kissing habits (Kort et al., 2014). These three spatial scales – within anatomic sites, among anatomic sites within a body, and among individuals – are then nested and potentially in interaction with each other.

We can also identify nested spatial scales in another example linked with plant-associated symbiotic fungi. A fraction of fungi recruited from the plant roots are transmitted to the other organs – leaves and seeds – of the plant (i.e., systemic distribution) (e.g., Vandenkoornhuyse et al., 2015), likely depending on the architecture and energy trade-offs of the individual. Fungi have recently been shown to disperse through plant-vegetative multiplication, colonizing young individual offsprings developing along the stolons (Vannier et al., 2018). But the fungal microbiota of a given individual plant is also influenced by the neighboring host composition (Bittebiere et al., 2020) and isolation from hosts of the same species at the centimetric scale (Mony et al., 2020b). At a larger scale, fungal spores and propagules can disperse over much longer distances, for instance with birds as vectors (Correia et al., 2019), thus at least in part, being under the influence of the macro-landscape scale. This example presents another illustration of a nested spatial scale structure, based on biotic landscapes constituted as host distribution, which likely affects microbial composition. In this example as for the first one, there is in addition a potential interaction between the landscape of microbes and the landscape of the hosts, to which microbes are associated. Disentangling the respective effect of each scale of landscapes in the microorganism distribution, and the dependency between microbial landscape and host landscape has not yet been done. Many interesting questions could then be raised among which the analysis of the respective effect of each spatial scale in shaping the distribution of microorganisms, the effect of the intensity of dispersal fluxes among scales, and their dynamics over time.

Considering time scales, few studies have demonstrated these nested scales, probably because studies that investigate the impact of temporal changes in landscape structure on microbiota are rare. However, there are many cases where such nestedness among temporal scales can be assumed, especially when a landscape is based on host distribution, and hence host phenology. To go even further, it is likely that both spatial and temporal multi-scales interact, making it even more complex to address these processes.

### Feedback Loops Within Microbial Landscapes

Landscape ecology generally analyzes how landscape structure shapes species distribution and abundance, assuming that there is no reverse effect. Yet, in contrast to macroorganisms, the activity of microorganisms is likely to reshape the structure of their own landscape. We can cite three examples of such potential feedback. The first example is linked to the huge role played by microorganisms in soil chemistry and structure (e.g., Bardgett and van der Putten, 2014). Decomposition of organic matter, as well as many biological cycles, are linked to bacterial or fungal activity (e.g., Schimel and Schaeffer, 2012). The patchiness of microorganisms in the soil may then lead to further changes in environmental patchiness, thereby affecting future generations of microorganisms in their foraging activity and dispersal. The second example is the microbial communities forming biofilms, groups of surface-adhering or free-floating cells, a case where free living microorganisms are interacting with each other to form a new environment and ecological habitat. These self-organized biofilms are mediated by interaction networks, which makes a feedback consequence on nutrient fluxes and spatial structure of the biofilms themselves (Nadell et al., 2016), social interactions and cross feeding which can modify microbial population spatial structure (West et al., 2007; Mitri et al., 2011; Mas et al., 2016). This cross feeding and cooperation among biofilm-members is supposed to be key for the biofilm stability and is likely a consequence of evolution of metabolic dependencies and specialization leading to a steady state among microbial populations (Mas et al., 2016). The third example is linked to biotic landscapes (Box 1). The interplay between microorganisms and all the biological functions of their hosts, i.e., growth, behavior and reproduction, affect their fitness (e.g., Rosshart et al., 2017). In the host-pathogen system, pathogen colonization of hosts may cause a drastic change in their host physiology and even their death. The way microorganisms are distributed among host patches is then likely to contribute to host population dynamics. Host patches could disappear, increase, or even move in case of mobile hosts, under the action of microorganisms, thereby modifying the spatial structure of the biotic landscape. This feedback, which is generally analyzed at the patch level (local conditions or host level), has not been investigated at the scale of multi-patches (i.e., landscape scale) and should be considered as a key particularity of microorganisms compared to macroorganisms.

## Effects of Landscape Mosaic Heterogeneity and Habitat Fragmentation on Microorganisms

### Landscape Spatial Heterogeneity

Studies that investigate the effect of landscape heterogeneity on microbiota are generally focused on analyzing the effect of composition or configuration at the scale of a given habitat patch, rather than investigating the effect of heterogeneity as a whole. This is done by concentrating on focal patches and then accounting for heterogeneity in the close neighborhood to the microbial assemblage under study (Figure 3).

#### Heterogeneity of Composition

Composition can be assessed while taking other types of elements in the landscape into account: for instance, the composition of the fungal microbiota associated with trees was shown to depend on the composition of plant species in the vicinity (Bogar and Kennedy, 2013), each plant species representing a particular habitat. The heterogeneity of composition can also be due to genetic differences within a host. Decreasing the frequency of susceptible host genotype compared to resistant ones in the landscape mosaic decreased the spread of a bacterial leaf streak in wheat (Mundt et al., 2011).

#### Heterogeneity of Configuration

Similarly, the impact of spatial configuration on the spread of pathogens at the landscape scale has been demonstrated with *Leptosphaeria maculans* (Bousset et al., 2018) that causes “blackleg” disease in canola (*Brassica napus*) and with the fusiform rust *Cropartium quercuum* in pine plantations (Perkins and Matlack, 2002). In both cases, the proximity of more susceptible stands of hosts and the absence of barriers to dispersal of the pathogen, such as non-host plants or particular land-use types facilitated the spread of the disease. The density and proximity of other particular landscape elements, such as roads, may also be important. Roads facilitate access to their host by the pathogens because roadsides are mowed regularly promoting pathogen spread (Laine and Hanski, 2006) but also because it is dispersed through the movement of cars or animals. For example, Laine and Hanski (2006) showed that *Plantago lanceolata* and its wind-dispersed obligate pathogen *Podosphaera plantaginis* were dispersed by the currents of air created by cars, and Jules et al. (2002) showed that the exotic root pathogen *Phytophthora lateralis* spreading on *Chamaecyparis lawsoniana* was dispersed by mud transported via vehicles and on people’s feet and animals’ hooves. Overall landscape configuration has thus mostly been seen as a driver of microorganism dispersal while other mechanisms, such as supplementation or complementation processes (Dunning et al., 1992) have not yet been studied.

### Landscape Habitat Fragmentation

Landscape effects are also linked to fragmentation, which includes both the effect of the reduction in habitat amount and/or the increase in isolation of habitat patches (Fahrig, 2003). The reduction in habitat amount affects species ability to survive and develop, due to an increase in extinction rate and the habitat’s limited carrying capacity. At the patch level, small patch size increases the effect of patch edges. Edges are indeed at the center of active exchange of energy, matter, and species from one patch to another. They may act as barriers or filters to the movement but also contribute to changes in abiotic conditions (nutrients, microclimatic conditions) inside the patches (Saunders et al., 1991; Murcia, 1995). These effects may be beneficial or detrimental to species, depending on the species’ ecological requirements. Isolation limits an organism’s ability to disperse and to colonize other patches in the landscape. Because of these effects, fragmentation is assumed to reduce biodiversity by increasing the susceptibility of species to environmental stochasticity leading to an increased risk of extinction (Fahrig, 2003). A wide range of studies deals with the effects of habitat fragmentation on microorganism assemblages, the effect either of habitat size or of habitat isolation, or both. In the case of biotrophs, the host determines the available habitat. Fragmentation is linked to the size of the host population and its distribution across the landscape, yet few studies have tried to disentangle the respective effects of patch size, isolation and edges. The combination of small and isolated patches generally increases the prevalence of pathogens, including fungal infections in plants (Groppe et al., 2001; Colling and Matthies, 2004), although the reverse effect has also been found, for example, Linert and Fischer (2003) reported higher prevalence of the fungal *Urocystis primulicola* on the plant *Primula farinosa* in fragmented landscapes.

#### Patch Size

The effect of habitat size has been widely studied but contrasted patterns have been demonstrated even within the same taxonomic group. Penttila et al. (2006) found that species richness and wood-decay fungi increase rapidly with an increase in area. On the other hand, no particular effect of habitat size was found to determine the composition of spores of arbuscular mycorrhizal (AM) fungi in forest soils (Mangan et al., 2004), whereas the colonization intensity of AM fungi was positively correlated with the size of the forest fragment (Grilli et al., 2012). The spread of pathogens has been associated with the presence and amount of edges. For instance, fungi were reported to colonize the leaves of woody seedlings three times faster in edge plots than in interior plots, perhaps due to interactions with damage caused by herbivory (Benitez-Malvido and Lemus-Albor, 2005).

#### Habitat Isolation

Habitat isolation is related to the distance between neighboring habitats (Box 1). The effects of isolation were originally studied by investigating the effects of geographic distance on community composition following the biogeography theoretical framework. Distances can range from one meter to the continental scale (Fierer and Jackson, 2006). First focusing on pathogens, research on habitat isolation has accumulated evidence that geographical distances among hosts determines the severity and incidence of disease, and likely also affects its spread (Thrall et al., 2003; Laine and Hanski, 2006). One of the very first works to investigate the effect of isolation at the community scale demonstrated a 50% decrease in ectomycorrhizal fungi richness associated with individual *Pinus* trees located at a distance of 1 000 m from the forest edge (Peay et al., 2010). This decrease was likely driven by dispersal-limitation mechanisms as demonstrated by Peay et al. (2012) who used a trap experiment and showed that the quantity and richness of spores of ectomycorrhizal fungi in the trap, and their colonization of sterile pine seedlings decreased rapidly with increased spatial distance from the host vegetation. Conceptual development in landscape ecology considers that the isolation effect is not only driven by geographic distance, but also by landscape structure (i.e., using patch-matrix or landscape mosaic model, Figure 2). By accounting for how landscape can facilitate or impede the dispersal of organisms (Taylor et al., 1993), landscape connectivity is suggested to be a key component of isolation metrics, even in microorganism studies. One simple metric used is the distance to the nearest patches of similar habitat. For instance, Vannette et al. (2016) demonstrated that fungal species composition associated with the tree, *Metrosideros polymorpha*, was more similar among highly connected habitat patches, i.e., patches with a habitat of same type in the close vicinity, than among poorly connected ones, i.e., patches with the same type of habitat located far away. A study by Peay et al. (2010) demonstrated the importance of particular fungal reservoirs as the composition of ectomycorrhizal fungi associated with *Pinus muricata* isolated trees embedded in a non-forested matrix, depended on the distance to large forest patches but not necessarily to the nearest isolated tree. These results suggest an effect of population size or age in the connectivity effect. On the contrary, connectivity is also determined by the existence of barriers to dispersal: for instance, in polar environments, the occurrence of mud boils due to frost was shown to modify microbial co-occurrence networks in bacteria in the soil (Ferrari et al., 2016). This study unexpectedly demonstrated that patches isolated through these barriers harbored higher species richness, probably due to a sheltering effect from the predators. Interestingly, these results suggest that biotic interactions may interplay with the connectivity effect and should thus be taken into account in future studies.

At a much smaller scale, for instance, in human microbiomes, the concept of connectivity within the body is still in its infancy, even though many observations support the validity of the concept applied to the distribution of microorganisms. For instance, the microbiota in the mouth, nose and stomach were shown to resemble each other more than they resembled lung communities, suggesting that the esophagus acts as a corridor that promotes microorganism dispersal (Bassis et al., 2015). The role of other components as corridors has been suggested as symptomatic patterns associated with diseases, such as the connection between nasal canal and the middle ear through the Eustachian tube that facilitates the spread of the bacterial agents of otitis (Chan et al., 2016). At an even smaller spatial scale, in the lung, microorganism community richness has been found to be a function of increasing distance to the supraglottis, seen as a reservoir of microorganisms (Dickson et al., 2015). Other studies suggest that microbial dispersal along corridors might be related to fluids like mucus or saliva. For instance, the velocity of the salivary film and the position of the teeth were reported to control the microbiota present on teeth and their susceptibility to be colonized by caries-associated bacteria (Proctor et al., 2018). The nasal mucus present in the nasal cavity transported microorganisms to the paranasal sites, together with an input of nutrients (Abreu et al., 2012; Aurora et al., 2013). In these particular studies, connectivity is mostly linked to the occurrence of a physical connection associated with a fluid vector (a corridor viewed as a conduit), which is a restricted case study of landscape connectivity.

## Dispersal and Metacommunities Within the Landscape Ecology Framework

### Dispersal for Microorganisms and Interactions With Landscape Parameters

Microorganisms can disperse either passively or actively. While some taxa disperse over long distances, others only disperse over very short distances, generating non-random distributions. Species also display different modes of dispersal leading to a wide range of dispersal distances, for instance, fungi can disperse at the centimetric scale through expansion of the vegetative mycelium but also at much larger spatial distance through aerial dispersal of spores. In addition, the dispersal of microorganisms often depends on the vector involved, wind (Allen et al., 1989), water, or host movement. Inside the human body, microorganisms often disperse in mucus (Proctor and Relman, 2017).

Microorganism dispersal can thus be indirectly linked to landscape characteristics through their effect on the vector. For instance, microbial dispersal, and especially pathogens’ has been shown to strongly depend on particular layouts of air conditioning ducts in public buildings (Fernstrom and Goldblatt, 2013). When it comes to dispersal mediated by hosts, the distribution and movement of microbes across the landscape is also tightly linked with the response of their host to the landscape structure. For instance, proximity to cattle and to urban zones modifies the behavior of wildlife and affects the spread of antimicrobial resistance, presumably due to effects on microbial distribution (Arnold et al., 2016). In a biogeography study of public restrooms, the composition of microbiota sampled on open surfaces, and its origin in the human body (annal, vaginal or skin) depended on the behavior of the restroom users, suggesting that the restroom can be considered as a landscape made up of different elements corresponding to different ecological uses (Flores et al., 2011). In plants, the propagation of microorganisms associated with seed dispersal is less well known, especially because only a small part of the plant microbiota can colonize and be transmitted by seeds (Shade et al., 2017). Although the effect of landscape characteristics, particularly connectivity, on seed dispersal has been demonstrated in many ecosystems (Uroy et al., 2019), their consequences for microorganism dispersal have just started being demonstrated (Correia et al., 2019).

### Metacommunity Structure in Microorganisms: Toward Landscape Explicit Consideration?

From a biogeographic perspective, many species can be structured as metapopulations (Hanski, 1994) in which distinct local populations are assumed to be linked by dispersal fluxes. This concept has been extended to the concept of metacommunities that simultaneously considers the role of species interaction (Leibold et al., 2004; Leibold and Chase, 2018). Until recently, four main models have been described depending on how species respond to local environmental conditions, dispersal limitation and disturbances (species sorting, patch dynamics, mass effects and neutral dynamics). This conceptual framework can be applied at a wide range of spatial scales. Although many authors have considered the individual components of the theory including the effect of local factors or interactions between species in shaping assemblages, there has been relatively few works done to organize this within a unified framework (Christian et al., 2015; Miller et al., 2018; Langenheder and Lindström, 2019).

Langenheder and Lindström (2019) review the literature for non-host associated microbes and conclude that environmental heterogeneity (influencing the sorting of species among habitat types) is very generally important. However they also review studies showing that dispersal limitation (large-scale distance effects), dispersal excess (small-scale distance effects), priority effects in species interactions (independent of environment), and stochasticity (including apparent neutrality or near-neutrality) are evident in different systems under different conditions. They also show that these effects are linked to other community and ecosystem attributes such as overall productivity, stability and scale effects, but that all of these effects vary, often inconsistently, among studies in ways that are still unresolved. Some of these effects have been additionally demonstrated more rigorously by manipulative experiments (e.g., Thrall et al., 2003; Livingston et al., 2013; Berga et al., 2015). Other effects that have been identified but not extensively studied include interactions with “macrobes” (e.g., Verreydt et al., 2012). Similarly, Miller et al. (2018) evaluate how metacommunity ecology can inform (and be informed by) the study of host-associated microbiomes. They also conclude that there is evidence of the same set of metacommunity processes as were found in non-host associated microbes, including habitat (i.e., host types) heterogeneity, dispersal limitation, priority effects, and stochasticity, and that the importance of these effects can be highly context dependent in ways that are not fully resolved. However, they also highlight the additional importance of host-microbiome feedback as potentially important factors to incorporate into metacommunity ecology. One set of mechanisms that have yet to be adequately addressed include the role of local genetic evolution that may also be responsible for legacy effects (Urban et al., 2008; Bahl et al., 2011; Vass and Langenheder, 2017) and these may be particularly relevant in microbes due to their large population sizes and short generation times (Miller et al., 2018).

Nevertheless, even though there are clear conceptual connections between them, the link between metacommunity and landscape ecology remains poorly resolved (Almeida-Gomes et al., 2020). To a large degree, this is because metacommunity ecology has focused more on species attributes and how they contribute to community assembly than to site attributes. A promising step toward reconciling the two involves the modification of joint species distribution models and related methods (primarily focused on the distribution of species) to address landscape distributions (e.g., Fournier et al., 2017; Leibold et al., 2020).

## Biotic Interactions Within the Microbiota and Evolutionary Effects on the Microbial Landscape

Landscapes for microbes can be shaped by a feedback process due to microbes’ distribution and activity, and to their biotic interactions. Below, we review different examples of such interactions that can influence landscape structure and its dynamics.

### Competition

If at the microscale, landscape patches of nutrients are ephemeral in space and over time, as is the case in aquatic environments (Yawata et al., 2014), these transient patches likely lead to high heterogeneity in microbial communities. This will be even more the case when foraging behavior differs among microorganisms, some producing a biofilm (a multicellular bacterial community embedded in an extracellular matrix) while others explore more patches (free living cells). Short term changes in micro-landscapes could then explain the fine-scale ecological differentiation of microbial communities (Yawata et al., 2014) as well as the temporal dynamics of meta-communities related to the dispersal tradeoff (i.e., forming a biofilm but promoting kin competition vs. high dispersion to limit competition although with the risk of not finding a new patch). As demonstrated by Yawata et al. (2014), these different behaviors and the associated tradeoff lead to population segregation at a small spatial scale. The same tradeoff could be expected in all microbial communities that form biofilms, possibly through enforcement processes related to cooperation (Agren et al., 2019) and where bacterial foraging could deeply affect meta-community changes and dynamics.

### Prey-Predator

In nature, microbial communities are also the subject of prey-predator relationships. A recent elegant study demonstrated that a protist species can strongly affect the spatial patterns of two other protist species it predated by prey sorting (i.e., prey preference), thereby affecting their response to the patches of resources that comprise the micro-landscape. This prey-predator relationship ultimately had a feedback effect on the distribution of the predator within the landscape (Hol et al., 2016; Livingston et al., 2017). Although to our knowledge, this topic has not yet been studied, microbial viruses can act in a similar way as protist predators by increasing microbial landscape complexity. For instance, the regulation of microbial populations density at macro-landscape scale has been demonstrated, with highly successful microorganisms being attacked by the proliferation of their specific viruses (i.e., “killing the winner hypothesis”) (Miki and Yamamura, 2005) leaving the habitat free for other less successful microorganisms. Because microbial predators regulate population size, microbial fitness decreases with an increase in its relative abundance. Thus, microbial predators do engineer the landscapes of bacterial communities but simultaneously depend on such landscape structure. Similar phenomenon was also demonstrated on biofilms. Grazing from protozoans was shown to shape biofilm volume and spatial heterogeneity (Bohme et al., 2009; Weeman et al., 2011).

### Mutualist Interactions

Symbiotic interactions among free-living members of the microbial community also drive the microbial community structure. Cooperative behaviors can emerge as an evolutionary process to escape competition with a member of the community that produces a shared public good while cheaters (i.e., those no longer able to produce it) become dependent on the producer and are fitter than the wild-type non-cheater (Morris et al., 2012; Mas et al., 2016). This evolutionary trajectory of dependencies through gene loss (e.g., Mas et al., 2016) can at least partly explain the complexity of co-occurring microbial communities and their spatial heterogeneity, but this evolutionary pathway also triggers a feedback process on the microbial landscape made up of the producer patches. In humans and animals, cellular disorder can induce or be induced by microbiota: members of the microbiota (pathobiome) can be involved in shaping inflammatory environments and in some cases could promote tumor growth and spread (Brennan and Garrett, 2016). Evolutionary processes can thus contribute to the spatial dynamics of both interactors at micro-scale and impact the fate and success of dispersal among patches of the microbial landscape.

### Bacterial Coexistence and Rapid Evolution

Diversity in communities is often viewed in light of ecological processes but, by modifying the microbial landscape, rapid evolutionary processes may also matter (Hart et al., 2019). To illustrate this issue, let us use the rapid evolution of resistance to antibiotics, which is among the best-documented cases of recent evolution in microorganisms (Nesme and Simonet, 2015; Hiltunen et al., 2017). The example of the evolution of antibiotic resistance can be summarized as a rapid evolutionary process of gene acquisition conferring new ecological abilities. As a result, the related eco-evolutionary processes modify the competitive hierarchy and in turn, may affect the coexistence outcome and realized niche. The dissemination of these new functional abilities will likely result in the rapid evolution of the microbial landscape. The eco-evolutionary processes that take place in the context of microbial landscapes is a fundamental frontier of knowledge that could be reached through a more holistic perception of the factors driving bacterial coexistence.

## Conclusion

The application of landscape ecology to the microbial world is still in its infancy despite an important set of works on microbial spatial ecology and biogeography. On the one hand, studies analyzing the effect of environmental heterogeneity on microorganisms have been for long developed without clearly using the concepts and methods of landscape ecology. On the other hand, the current research effort on landscape ecology for microorganisms is mostly focused on pathogens and disease risk assessment. These latter studies, despite their strong interest, may limit our knowledge on microbial landscape ecology toward specific host-pathogens systems, and methods used to investigate symptoms rather than species presence and abundance. We demonstrated, however, through this review an increasing interest to fill the gap between both approaches, and transpose concepts and methods of landscape ecology for analyzing the structure of microbial assemblages.

If most existing literature on the topic describes the landscape using the continuum landscape model (i.e., continuous environmental heterogeneity), there is an emerging set of works that use the patch-matrix model, i.e., consider landscapes as constituted of discrete favorable habitat patches. Integrating the landscape mosaic in the description of the microbial landscape is poorly done yet. However, there is an obvious interest of using the mosaic landscape model for microbes, especially because some of these microorganisms develop in biotic landscapes constituted of hosts (i.e., discrete habitat patches of different kinds), but also because microbial dispersal likely depends on the permeability of the landscape matrix. There is then a strong need to develop the dedicated metrics for using this conceptual model to microorganisms.

Through this review, we highlighted some convergences in organisms’ response to landscape features, among free-living organisms and microorganisms associated with plants, animals or humans. We demonstrated especially the importance of landscape configuration (and not only composition) as a driver of microbial community heterogeneity in space and time; and the key role of dispersal mechanisms - both active and passive - in this relationship. Some ecological processes and their influence differ however, for microorganisms compared to macroorganisms. Microorganisms’ small size and short generation time affect their responses to landscape characteristics. These responses can occur at a very small spatial scale, and across several generations promoting the importance of evolutionary processes in species assembly. The scale of effect for microorganisms is then more complex than for macroorganisms, involving potentially nested-spatial and time scales. These nested-scales depend on the dispersal abilities of microorganisms, and on microorganisms’ potential interactions with a host or with other microbes. Studying landscape ecology of microbes should then involve sampling or experimental designs across multiple scales. In host-associated microorganisms, it should also take into account the “host-microbes” system as a whole, for designing the study and in interpreting the results. Another important point is the existence of feedback effects of microbes on their own landscape. Thanks to their distribution and activity, microorganisms modify the abiotic conditions, or act on their host fitness and behavior. They shape then their future landscape. Macroorganisms affecting local environmental conditions at the patch scale is a well-known feedback; we demonstrated here that it could be up scaled at the landscape level for microorganisms. The existence of this landscape feedback effect opens a large array of hypotheses on its influence on the metacommunity internal processes, and on a possible coevolution of microbes with their landscape.

These specificities listed above may then call for further developments on the theoretical framework of landscape ecology for microbial organisms. Such development may overall help to reach a comprehensive view of stochastic and deterministic processes in their assembly, and develop approaches that are more functional. Because of their pivotal role in many ecosystem services, from health to food production, the development of landscape ecology for microorganisms should have major consequences for our understanding of their assembly and potentially for their manipulation in anthropogenic and natural ecosystems.

## Author Contributions

All authors discussed about the content of the article and reviewed the draft, and gave final approval for publication. CM and PV wrote the first draft of the manuscript with inputs from ML, BB, and KP.

## Conflict of Interest

The authors declare that the research was conducted in the absence of any commercial or financial relationships that could be construed as a potential conflict of interest.
